# Targeting Ribonucleases with Small Molecules and Bifunctional Molecules

**DOI:** 10.1021/acschembio.3c00191

**Published:** 2023-06-29

**Authors:** Lydia Borgelt, Peng Wu

**Affiliations:** Chemical Genomics Centre, Max Planck Institute of Molecular Physiology, Otto-Hahn-Str. 11, Dortmund 44227, Germany; Department of Chemical Biology, Max Planck Institute of Molecular Physiology, Otto-Hahn-Str. 11, Dortmund 44227, Germany

## Abstract

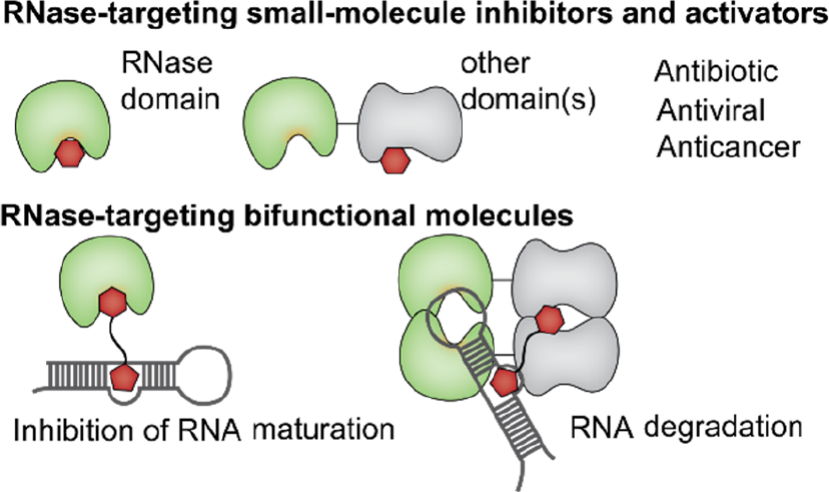

Ribonucleases (RNases) cleave and process RNAs, thereby regulating the biogenesis, metabolism, and degradation of coding and noncoding RNAs. Thus, small molecules targeting RNases have the potential to perturb RNA biology, and RNases have been studied as therapeutic targets of antibiotics, antivirals, and agents for autoimmune diseases and cancers. Additionally, the recent advances in chemically induced proximity approaches have led to the discovery of bifunctional molecules that target RNases to achieve RNA degradation or inhibit RNA processing. Here, we summarize the efforts that have been made to discover small-molecule inhibitors and activators targeting bacterial, viral, and human RNases. We also highlight the emerging examples of RNase-targeting bifunctional molecules and discuss the trends in developing such molecules for both biological and therapeutic applications.

## Introduction

**Figure 1:**
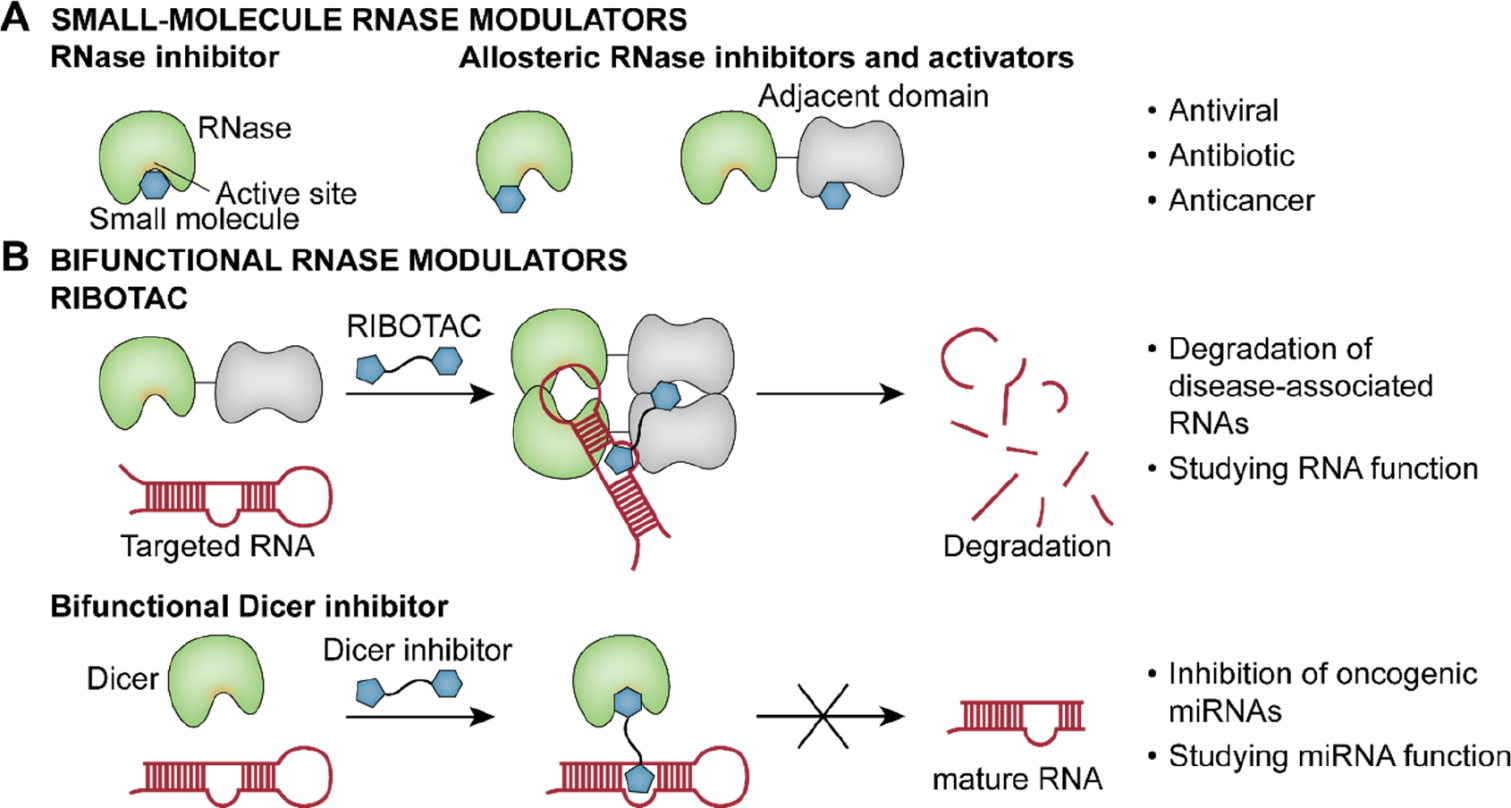
Current RNase-targeting strategies using small molecules and bifunctional molecules. (A) Small-molecule modulators include inhibitors that bind to the RNase active site, inhibitors and activators that bind to allosteric sites, or binders to adjacent domains. (B) Described bifunctional modulators enable targeted RNA degradation by the recruitment of RNase L or inhibition of Dicer-mediated RNA cleavage by coupling a weak Dicer inhibitor to an RNA binder.

Ribonucleases (RNases) are RNA-cleaving proteins that regulate the metabolism of RNAs. The two main classes of RNases are endoribonucleases and exoribonucleases. In humans, RNases belong to a superfamily constituted of at least 122 proteins that not only act as simple RNA degraders but are also involved in essential nondigestive cellular functions involved in immune response, RNA maturation, and angiogenesis.^1−3^ Distinction of these processes relies on the differences in RNA-substrate sequence of RNases and specificity for single- or double-stranded RNAs. RNases either function in a processive manner or cleave their substrate only once after binding.^4^ The nucleases are master regulators of RNA-dependent pathways and play indispensable roles in RNA biogenesis.^5,6^ Therefore, small molecules modulating RNase activities are useful tools for studying the regulatory mechanisms involving both human and pathogenic RNases and are potential candidates for the development of therapeutics. Representative examples of such small molecules include inhibitors of viral RNases influenza polymerase acidic (PA) endonuclease and human immunodeficiency virus (HIV) RNase H, and several human RNases. The potential of RNases as therapeutic targets was discussed in a previous opinion article;^1^ however, a review summarizing the scattered examples of these RNase-targeting small molecules is currently missing. To note, in addition to small molecules, RNases have been targeted by the emerging class of proximity-inducing bifunctional molecules for various chemical biology applications. Therefore, in this Review, we highlight two aspects of the current landscape in RNase targeting: small molecules for the development of therapeutics and bifunctional molecules that modulate RNA biogenesis and metabolism (Figure 1). The former aspect is divided into three target families, bacterial RNases, viral RNases, and human RNases. We also discuss trends and future directions to explore RNases as drug targets for small and bifunctional molecules.

## SMALL-MOLECULE INHIBITORS OF BACTERIAL RNASES

### RNase P

**Figure 2:**
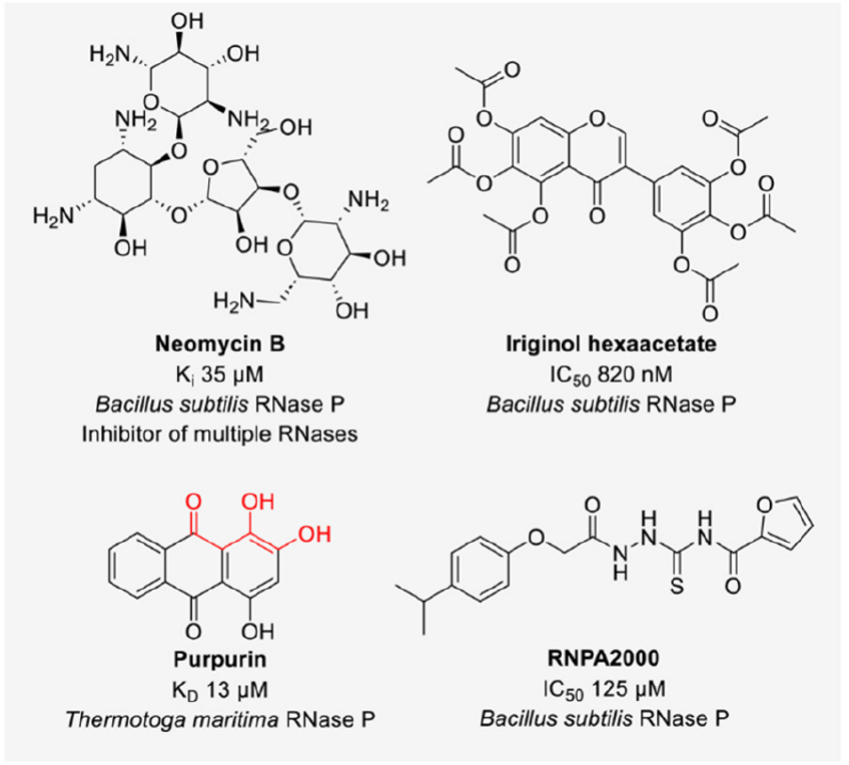
Small-molecule inhibitors of bacterial RNase P (the red color indicates the metal-chelating pharmacophore of purpurin).

Bacterial ribonuclease P (RNase P) is a unique RNase since it is a ribonucleoprotein functioning as a ribozyme. RNase P catalyzes the maturation of tRNA (tRNA) by the cleavage of the 5′ end of precursor tRNA. Inhibitors of RNase P enzyme function, which is crucial for bacterial survival, could potentially serve as antibiotics. The only FDA-approved inhibitors of RNase P to date are antibiotic aminoglycosides such as neomycin. To note, RNase P inhibition is, however, not the sole mechanism of action for aminoglycoside antibiotics since their interaction with rRNA is known to cause errors in translation.^7^ The development of fluorescence real-time tRNA cleavage assays enabled increased throughput screening for inhibitors.^8,9^ A fluorescence polarization assay using a fluorophore at the 5′-end of the precursor tRNA confirmed neomycin B as an RNase P inhibitor (Figure 2). A screening of 2880 compounds discovered iriginol hexaacetate that inhibited RNase P with an IC_50_ of 0.8 μM (Figure 2).^8^ A Förster resonance energy transfer (FRET) assay-based screening retrieved purpurin as an RNase P inhibitor (Figure 2). Purpurin bound to RNase P with a *K*_D_ of 13 μM and its binding to the protein component of the enzyme was confirmed by crystallization of a purpurin-RNase P complex.^9^ Purpurin carries a three-oxygen pharmacophore that was often observed to coordinate the divalent metal ions of RNases.^9,10^ However, data on the antibacterial effect of purpurin was not reported.^9^ Additionally, RNPA2000 was reported to show weak RNase P inhibitory activity (IC_50_: 125 μM, Figure 2).^11^ A concern for the identified RNase P inhibitors, including RNPA2000, iriginol hexaacetate, and purpurin, was that they acted as aggregators and are likely unspecific RNase P inhibitors.^12,13^ In addition to small molecules, a FRET assay was employed to evaluate rationally designed and modified oligonucleotides as inhibitors of RNase P. Antisense oligonucleotides targeting the RNA component of RNase P were coupled to cell-penetrating peptides to yield conjugates that inhibited bacterial growth. The best-performed conjugates showed IC_50_ values of ∼100 nM, being the most potent RNase P inhibitors reported to date.^14^

### RNase E

RNase E is another bacterial RNase that functions as an endonuclease involved in ribosomal maturation and RNA turnover. RNase E is a central component in the formation of the degradosome complex in *E. coli*, and small-molecule inhibitors were identified with the aim of studying the cellular functions of RNase E. A virtual screening predicted small-molecule binders of RNase E, which were evaluated by biochemical assays and SPR, albeit with weak millimolar activities.^15^ In summary, although inhibitors against the bacterial RNases such as RNase P and E are potential antibiotics, no potent and selective small-molecule inhibitors have been described so far. Even for the reported inhibitors listed here, their effects in inhibiting bacterial growth mainly were not reported or investigated.

## SMALL-MOLECULE INHIBITORS TARGETING VIRAL RNASES

Small molecules have been reported for viral ribonucleases as antiviral therapeutics, such as those targeting HIV and HBV RNase H, influenza virus PA endonuclease, and the RNases nsp-14-ExoN and nsp15 of SARS-CoV-2.^16−18^

### RNase H

RNase H is part of viral reverse transcriptase that removes RNA template strands to allow the synthesis of double-stranded DNA from viral genomic RNA, leading to the incorporation of the double-stranded DNA into the host genome by integrases.^19,20^ To date, HIV is treated by a combination of drugs in highly active antiretroviral therapy (HAART) and all HIV enzymatic activities except RNase H can be targeted effectively.^21,22^ Despite numerous studies aiming to identify RNase H inhibitors, current FDA-approved HIV-1 reverse transcriptase inhibitors all target the polymerase activity, but not the RNase H activity of the enzyme.^23,20^ RNase H has been extensively studied as a drug target already since the 1990s, as reviewed recently by Tramontano et al.^16,24−26^ Screenings for RNase H inhibitors were historically performed by denaturing gel-based assays with isotope-labeled RNA substrates in limited throughput.^24,25,27^ More recent studies used fluorescent assays to identify active compounds, e.g., a FRET assay using an RNA-DNA hybrid in which the DNA was labeled at both ends with a fluorophore quencher pair. Hydrolysis of the RNA led to DNA-hairpin formation and induced fluorescence quenching.^28^ Another assay used a fluorophore-labeled DNA annealed with a quencher-labeled RNA, leading to a fluorescent signal upon RNA cleavage.^29^

**Figure 3:**
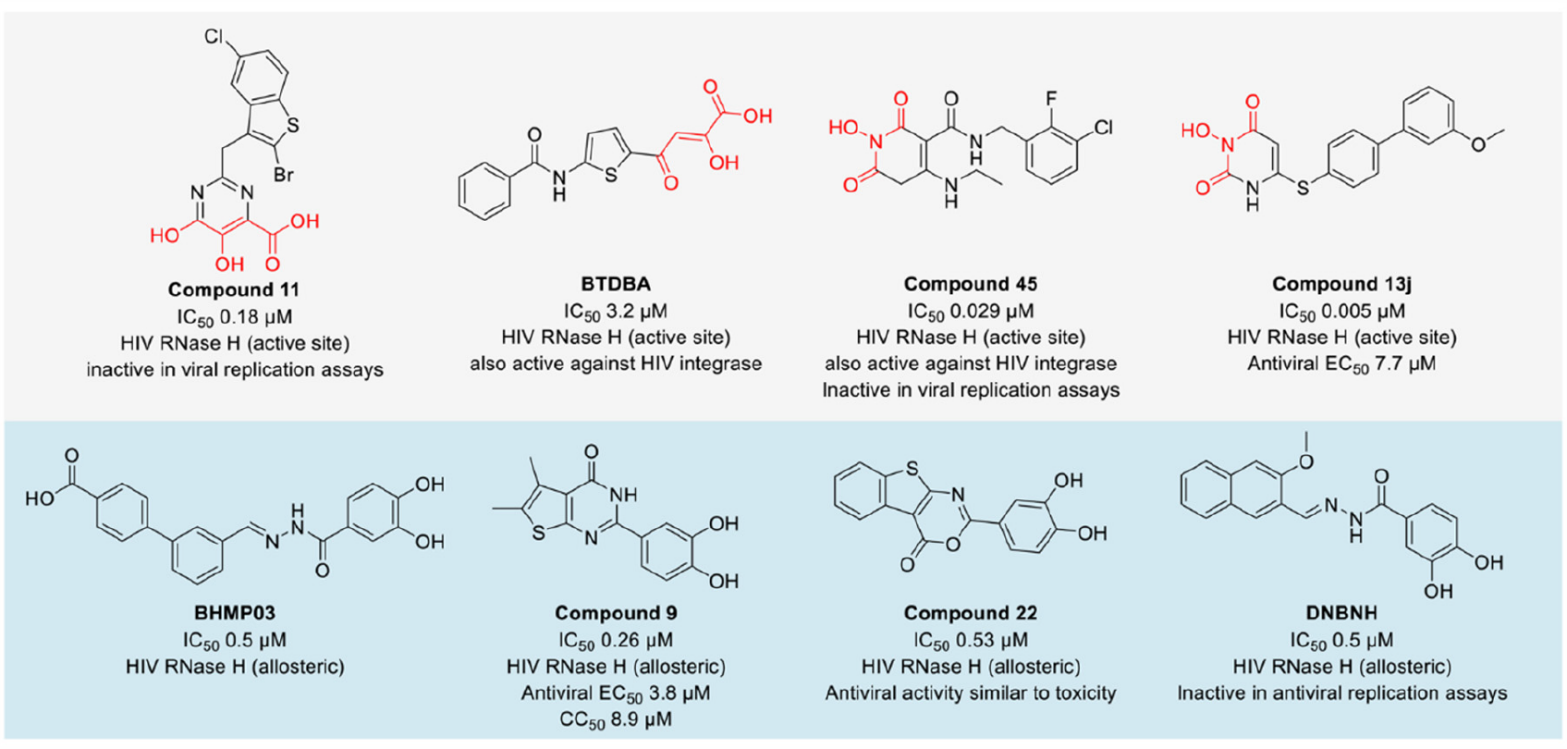
Structures of inhibitors of HIV RNase (the red color indicates metal-chelating pharmacophore).

In general, three classes of RNase H inhibitors were described, metal-chelating active-site binders, allosteric inhibitors, and dual inhibitors against both reverse transcription and RNase activities.^16^ Active-site binding molecules mostly carry a three-oxygen pharmacophore that allows chelation of the magnesium ions of the DEDD (Asp-Glu-Asp-Asp) family endonuclease RNase H. Small molecules with diverse scaffolds have been identified as active-site inhibitors and evaluated in structure–activity relationship studies to optimize affinities (Figure 3). Most molecules were reported to show low micromolar IC_50_ values in biochemical assays, with some showing nanomolar potency.^16^ Several molecules, such as the pyrimidinol carboxylic acid **11** that showed an IC_50_ of 0.18 μM against HIV RNase H domain did not inhibit viral replication in HIV infectivity assays.^30^ Complex structures of HIV RNase H domain and small molecules have been solved and clearly showed the binding mode involving the three-oxygen pharmacophore interacting with the metal ions.^16,31−33^ The RNase H domain active site is a shallow pocket difficult to be drugged without metal chelation.^16^ Active-site analysis revealed homology to the catalytic center of HIV integrase that contains two divalent metal ions and is responsible for inserting viral DNA into host genomic DNA.^34^ Modification of integrase inhibitors led to the identification of dual inhibitors of RNase H and HIV integrase, such as one of the first described RNase H inhibitors, 4-[5-(benzoylamino)thien-2-yl]-2,4-dioxobutanoic acid (BTDBA).^35,36^ Dual inhibitors against two enzymatic functions of the same virus boosted the activity of the molecules against HIV replication and thus were further investigated.^36^ One of the most potent HIV RNase H domain inhibitors reported to date is the *N*-hydroxypyrimidinedione **45** with an IC_50_ of 25 nM against RNase H and an IC_50_ of 21 nM against HIV integrase. Compound **45** showed an EC_50_ of 15 nM in an antiviral replication assay.^37^ Another promising RNase H inhibitor is the *N*-hydroxypyrimidinedione **13j** with an IC_50_ of 5 nM against RNase H, ∼1000-fold less activity against HIV integrase (IC_50_ 4 μM), and an EC_50_ of 7.7 μM in the antiviral replication assay. The large difference in biochemical and cellular activities could be explained by high substrate abundance and the fact that small molecules could only bind to the RNase H domain while substrate binding is highly dependent on its interaction with the polymerase domain. Thus, it is a challenge for small molecules to outcompete the substrates to achieve potent inhibition.^38,39^ Active-site inhibitors of HIV RNase H were tested for their inhibitory activity against HBV RNase H, given that the HIV and HBV RNase H share 23% amino acid sequence homology. Among compounds that showed moderate activity, the *N*-hydroxypyridinediones were investigated in a further SAR study.^28,40^ Other reported HBV RNase H inhibitors either showed weak inhibitory potency or suffered from cytotoxicity.^40−42^

While active-site RNase H inhibitors all carry a chelating-triad pharmacophore, inhibitors without this motif were characterized by crystallography and NMR as allosteric inhibitors. Among them was the acylhydrazone BHMP03 (IC_50_ 0.4 μM) binding to the substrate handle region of RNase H.^16^ Vinylogous ureas such as NSC727447 were identified as allosteric RNase H inhibitors by screening 230,000 natural compounds and led to the development of 3′,4′-dihydroxyphenyl-containing thienopyrimidinones (compound **9** and analogues) with submicromolar activity in biochemical assays and low micromolar activities in antiviral replication assays.^16,43^ Further SAR studies led to the identification of benzothienooxazinone compound **22** as a dual inhibitor of RNase H and reverse transcriptase activities of the HIV enzyme with IC_50_ values of 0.53 and 2.90 μM, respectively.^44^ Other dual inhibitors were based on the structure of the dihydroxy benzoyl naphthyl hydrazone DHBNH but did not show improved activity in comparison with that of compound **22**.^45,46^

### Nsp14 and Nsp15

**Figure 4:**
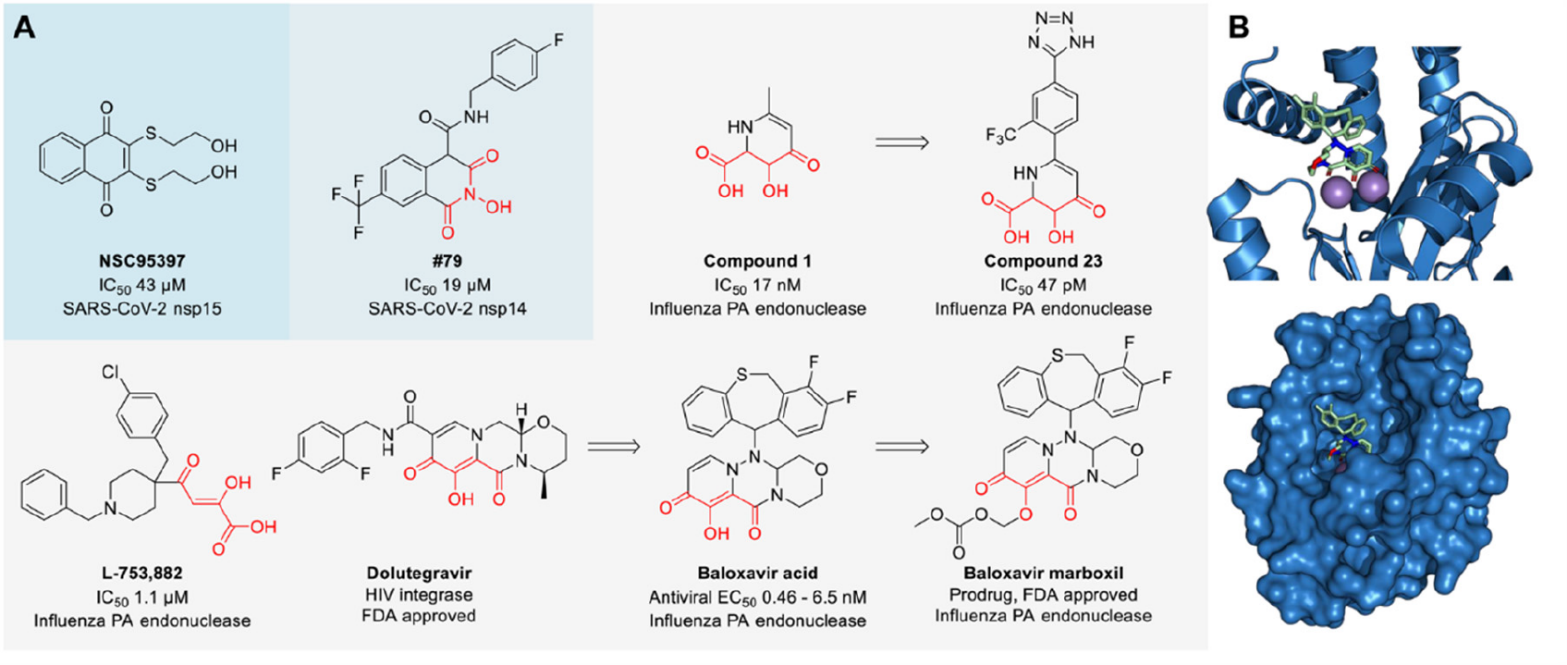
(A) Structure of small-molecule inhibitors of nsp14 and nsp15 of SARS-CoV-2 and PA endonuclease. The red-colored structure indicates the metal-chelating pharmacophore. (B) Crystal structure of baloxavir acid bound to the PA endonuclease of influenza A virus showing the interaction of the compound with the metal ions (top) and a surface representation (bottom) (PDB 6FS6).

Recently, the first inhibitors targeting SARS-CoV-2 ribonucleases nsp14 and nsp15 were reported. Nsp15 is an endoribonuclease cleaving RNA 3′ of uridines to prevent host recognition of the virus. Virtual screening of approved drugs and drugs under investigation for approval identified dutasteride and tasosartan as inhibitors of nsp15, but biochemical assays revealed only 40% inhibition at 600 μM concentrations.^17^ Further screenings led to the identification of kinase inhibitor NSC95397 as an inhibitor of ribonuclease nsp15 with micromolar activity but without effect in antiviral replication assays (Figure 4A).^47−49^ Uracil derivative tipiracil is a low-micromolar (IC_50_ 7.5 μM) nsp15 inhibitor with antiviral activity.^50^

Nsp14 is a proofreading exonuclease enhancing replication fidelity, rendering nucleoside analogues ineffective as drugs against SARS-CoV-2. Nsp14 inhibition was proposed as a strategy to enable nucleoside analogues to be active.^18^ First reports of nsp14 drug discovery efforts identified micromolar inhibitors from a FRET assay using fluorophore-labeled annealed RNA strands as a probe. Nsp14 was identified to belong to the DEDD superfamily of ribonucleases harboring divalent metal ions.^51^ Thus, it is unsurprising that one of the identified inhibitors, compound **79**, harbored the chelating triad pharmacophore as described for RNase H inhibitors (4).^52^ A SAMDI assay screening of 10,240 small molecules detecting RNA-cleavage by mass spectrometry led to the identification of an nsp14 inhibitor with an IC_50_ of 5.7 μM.^53^ Fragments with potential allosteric binding mode were discovered by fragment screening and could serve as a starting point for future nsp14 inhibitors.^54^

### PA Endonuclease

Another well-studied viral nuclease target is the influenza virus PA protein possessing endonuclease activity that was reported to be active against RNA and DNA substrates. It cleaves host-cell mRNA caps that are then used as primers for the transcription of viral mRNA.^55,56^ The enzymatic function is crucial for viral replication, which makes the influenza PA endonuclease a prime target for antiviral therapeutics. Like RNase H, PA endonuclease has two crucial divalent metal ions (Mn^2+^ or Mg^2+^) in its active site.^56^ Therefore, structures of reported inhibitors of influenza PA endonuclease are similar to inhibitors of viral RNase H. Nearly all inhibitors harbor a three-oxygen metal-chelating pharmacophore combined with hydrophobic moieties (Figure 4A).^57^ The metal-chelating pharmacophore for inhibition of the cap-snatching endonuclease was identified via screening already in the 1990s. One identified inhibitor was L-735,882 which inhibited influenza endonuclease with an IC_50_ of 1.1 μM.^58^ SAR exploration improved the activities (IC_50_ < 1 μM).^57^ Recently, a fragment-based approach starting with metal-chelating compound **1** led to the discovery of compound **23** which displayed an IC_50_ of 47 pm. However, the measured EC_50_ of compound **23** in the antiviral replication assay was in the low micromolar range.^59^ The most successful PA endonuclease inhibitor is baloxavir marboxil, which the US FDA approved in 2018 for the treatment of the influenza virus. Baloxavir was developed based on a rational design from the approved HIV integrase inhibitor dolutegravir (Figure 4), as the HIV integrase active site shares structural similarity with influenza PA.^60,61^ Baloxavir marboxil is a prodrug derived from the potent inhibitor baloxavir acid by shielding the hydroxyl group to increase bioavailability.^60,62,63^ Baloxavir acid had single-digit nanomolar or subnanomolar potency against 22 different influenza A and B strains and showed an improved effect in clinical trials compared with the results induced by the treatment of the neuraminidase inhibitor oseltamivir.^64^

Taken together, viral nucleases have been studied as antiviral drug targets for more than three decades. The influenza endonuclease inhibitor baloxavir is a representative example that has been approved for antiviral clinical usage. Besides influenza endonuclease, small molecules have been reported for the HIV RNase H, but still with limited potency in inhibiting virus replication, so RNase H remains the only HIV enzymatic function that has not been successfully addressed clinically. The Covid-19 pandemic promoted the study of RNases of SARS-CoV-2, and emerging inhibitors targeting nsp 14 and nsp15 are being reported.

## SMALL MOLECULES TARGETING HUMAN RIBONUCLEASES

Human ribonucleases are essential effectors in various cellular pathways responsible for immune responses, apoptosis, and inflammation. Thus, the modulation of human RNases with small molecules has been studied as a promising strategy for developing therapeutics and various biological applications. For example, RNase A family ribonucleases RNase 1 and Angiogenin are secretory enzymes involved in host defense, tightly regulated by RNase inhibitor protein.^65,66^ First reported RNase A family inhibitors were derived from nucleotide structures and showed nanomolar activities, followed by a few small-molecule inhibitors with moderate activities.^67−69^

### Dicer

The RNases Drosha and Dicer are central regulators of the maturation of noncoding RNAs. The enzymes specifically cleave primary and precursor miRNA transcripts to generate mature miRNAs. Both Drosha and Dicer are conserved processors of canonical miRNAs. No small-molecule inhibitor of Drosha has been reported to date. Small-molecule modulators of Dicer activity allow studying Dicer biology or serve as starting points for the development of anticancer agents. To identify Dicer inhibitors, a fluorescence quenching assay using double-stranded fluorophore- and quencher-labeled RNA substrate was developed to monitor Dicer activity, which identified the aminoglycoside kanamycin that inhibited Dicer processing by 40% at a concentration of 100 μM.^70^

### Caf1 and PARN

**Figure 5:**
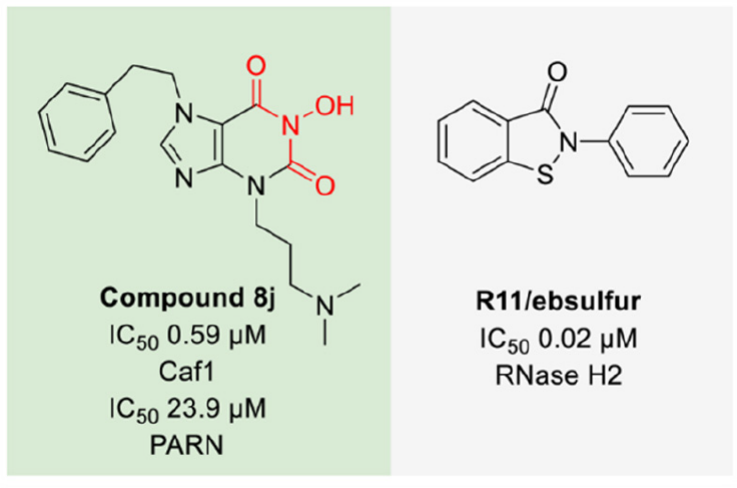
Structures of inhibitors of human RNases Caf1 and RNase H2 (the red color indicates the metal-chelating pharmacophore).

Ribonucleases involved in mRNA post-transcriptional regulation and turnover are mRNA deadenylases such as Caf1/CNOT7, which is part of the deadenylating Ccr4-Not complex, and Poly(A)-specific ribonuclease (PARN). Both enzymes have two divalent metal ions in the catalytic site coordinated by a DEDD motif. Caf1 inhibitors were discovered to develop probes to assist the biological understanding of the RNase. A FRET assay employing a fluorophore-labeled RNA probe was applied. The RNA probe hybridized with a fluorescently labeled DNA when it was not cleaved by Caf1.^71^ The most potent Caf1 inhibitors with low micromolar to submicromolar IC_50_ values were inspired by HIV RNase H inhibitors and harbor the metal chelating three-oxygen pharmacophore, such as compound **8j** that also showed activity against PARN (Figure 5).^72^ Other published PARN inhibitors are mainly nucleoside analogues and aminoglycosides.^73−76^

### RNase H2

The RNase H2 is a DEDD superfamily metalloenzyme that cleaves the RNA strand of DNA–RNA duplexes.^77^ RNase H dysregulation in humans is associated with the genetic autoimmune disease Aicardi-Goutières syndrome (AGS) that causes neurological dysfunction early during infancy.^78^ A screening of 47,520 compounds employing a fluorescent assay using fluorophore- and quencher-labeled RNA-DNA duplex substrates led to the identification of isothiazolidinone R11/ebsulfur with an IC_50_ of 0.02 μM against RNase H2 (Figure 5).^79^

### RNase L

**Figure 6:**
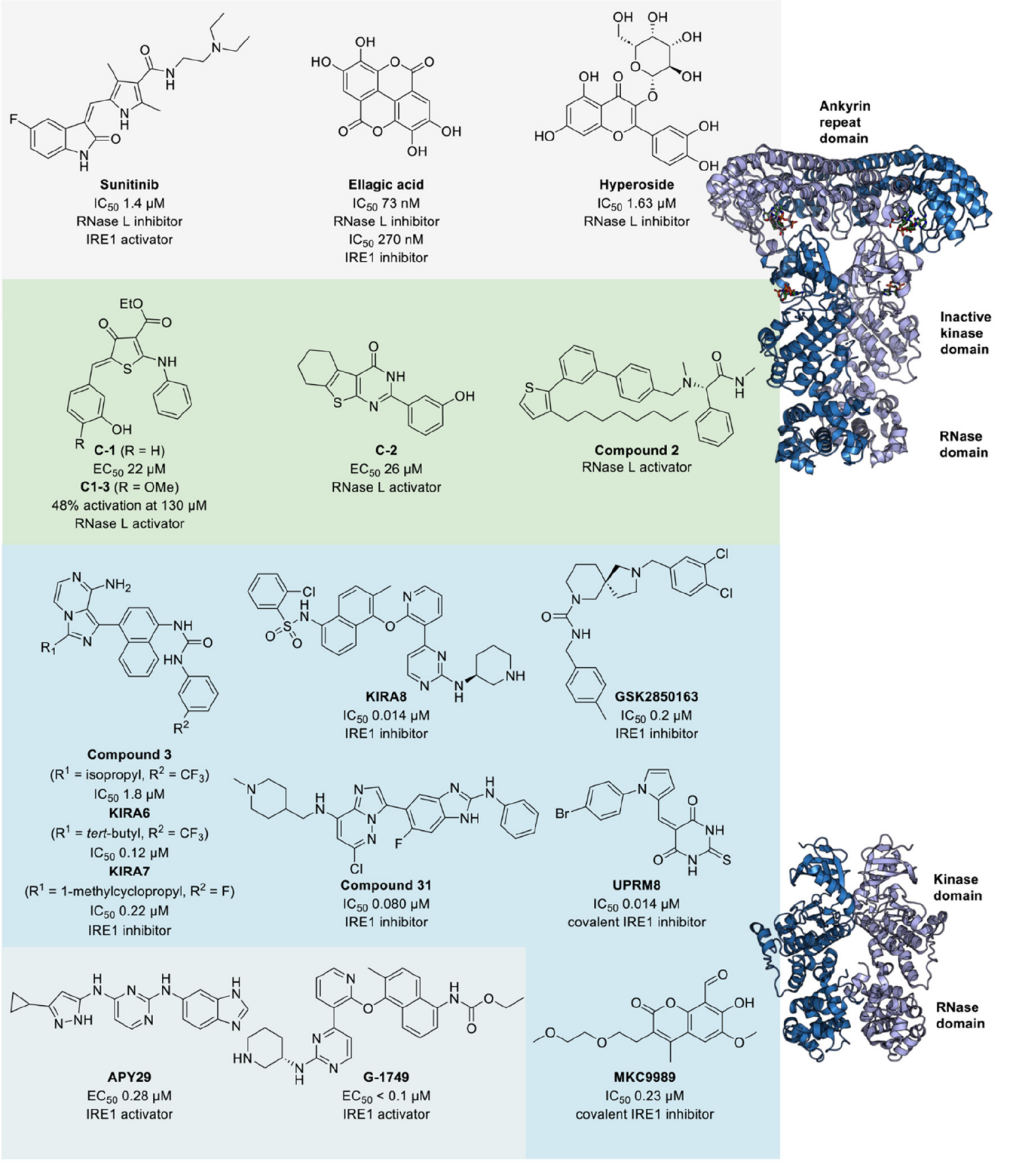
Small molecules targeting human RNases. RNase L inhibitors (gray background), RNase L activators (green background), and IRE1 modulators (blue background). The full-length structure of RNase L is shown (PDB 4O1P) together with the kinase and RNase domains of IRE1 (PDB 6W3C).

The latent ribonuclease (RNase L) is another human RNase implicated in AGS. RNase L is endogenously expressed in human cells and is central to innate immune and antiviral responses.^3^ The oligoadenylate synthase recognizes and binds double-stranded RNA upon viral infections and produces 2′-5′-linked oligoadenylates (2′-5′A).^80^ 2′-5′A bind to RNase L to induce dimerization to form the catalytically active, dimeric form of RNase L.^81^ Activated RNase L cleaves host and viral single-stranded RNA leading to a global translational arrest.^82,83^ RNase L consists of an *N*-terminal ankyrin repeat domain which is mainly responsible for 2′-5′A binding, a catalytically inactive kinase domain that binds ATP, and a C-terminal ribonuclease domain that cleaves the substrate RNA upon dimerization.^81^ Both small-molecule inhibitors and activators of RNase L have been reported (Figure 6). Inhibitors could serve as candidates for the treatment of AGS, while activators were developed as antiviral compounds.^84,85^ The kinase domain of RNase L shares high sequence homology with that of dsRNA-dependent protein kinase R (PKR) and inositol-requiring enzyme 1α (IRE1), and therefore, reported RNase L inhibitors share structural similarity of small-molecule kinase inhibitors that target the kinase domain, such as the FDA-approved receptor tyrosine kinase inhibitor sunitinib.^86^ Sunitinib was tested with varied IC_50_ values against RNase L ranging from 1.4 to 33 μM and showed an enhanced effect of oncolytic viruses against tumors in mouse models.^87−89^ The complex structure of sunitinib and RNase L confirmed the binding at the kinase domain and suggested dimer destabilization as the inhibition mechanism. Exchange of the fluorine substituent to chlorine improved the inhibitory activity by ∼4 fold.^89^ Screenings of 500 kinase inhibitors and 840 fragments using a FRET-based assay employing a single-stranded RNA probe labeled with a fluorophore quencher pair resulted in the identification of ellagic acid (IC_50_ 73.5 nM) and hyperoside (IC_50_ 1.63 μM) as RNase L inhibitors.^84,90^ The discovery of RNase L activators was based on similar FRET assays used to evaluate inhibitors, albeit without the addition of the natural activator 2′-5′A.^91−94^ First, thiophenone C1 and thienopyrimidinone C2 were retrieved as RNase L activators out of a library of 30,000 small molecules with EC_50_ of 26 and 22 μM, respectively, with a proposed activating mechanism by binding to the 2′-5′A-binding site.^91^ A more recent study optimized the thiophenone scaffold of C1 and yielded compound C1–3 that showed 48% activation at 130 μM compared with 2′-5′A at 100 nM.^92^ Combination of the 2-aminothiopheonone scaffold of C1–3 with that of the pyrrole scaffold of sunitinib led to RNase L inhibitors with a hybridized scaffold.^95^ An extensive SAR study focusing on the aminothiophene core scaffold and a screening of 240,000 small molecules resulted in small-molecule binders that stabilized RNase L but without significant improvement on the RNase L activating potency.^93,94^ Very recently, compound **2** was identified by DNA-encoded library-based screening as an RNase L activator that induced RNase L dimerization in micromolar potency.^96^

### IRE1

The serine/threonine-protein kinase/endoribonuclease IRE1 regulates the unfolded protein response located on the membrane of the endoplasmic reticulum (ER).^84^ IRE1 consists of an *N*-terminal ER-lumenal domain involved in the unfolded-protein detection, a transmembrane region, a kinase domain, and an RNase domain.^97^ Kinase and RNase domains are structurally related to those of RNase L, and the RNase domain fold is distinct from other proteins (Figure 6).^98^ Unfolded proteins induce dimerization of IRE1 and downstream signaling, including autophosphorylation and RNase activation.^97,99^ Activated RNase cleaves ER-bound RNA for its decay and specifically cuts X-box binding protein-1 (XBP1) mRNA in a nonconventional splicing mechanism, leading to the expression of a potent transcription factor.^86,100^ One reported downstream target of spliced XBP1 is the oncogene MYC. Thus, IRE1 activity is associated with cancer progression, aggressiveness, and poor prognosis.^101−104^ In addition to being studied as an anticancer target, IRE1 has also been implicated in diabetes and angiogenesis regulation.^105,106^ IRE1-targeting modulators were developed against the kinase and RNase domains (Figure 6).^86^ Similar to RNase L, reported kinase inhibitors were repurposed for IRE1 inhibition.^97,107^ Interestingly, some kinase inhibitors did not inhibit RNase function but activated IRE1 RNase activity instead.^106,108,109^ In general, type I kinase inhibitors stabilized the active kinase conformation and activated IRE1 RNase activity promoting oligomerization, while type II kinase inhibitors stabilized the inactive kinase conformation and inhibited RNase enzymatic activities.^107,110,111^ Assays for the identification of IRE1 modulators comprise splicing assays, fluorescence-based cleavage assays using fluorophore- and quencher-labeled XBP1 RNA substrate, and phosphorylation assays that focused on kinase activity instead of RNase activity.^107,109,112,113^ IRE1 inhibitor imidazo[1,5-α]pyrazine-8-amine compound **3** was identified based on screening of known type II kinase inhibitors, and further modification led to the development of compounds KIRA6 and KIRA7.^107,109,114^ Both KIRA6 and KIRA7 were potent inhibitors in biochemical and cellular evaluations, but a photoaffinity labeling approach revealed low selectivity of the imidazo[1,5-α]pyrazine-8-amine scaffold toward IRE1.^109,114,115^ The sulfonamide KIRA8 was an optimized hit with an IC_50_ of 0.014 μM in biochemical assays, but it did not affect cancer cell viability when screened against more than 300 different cancer cell lines.^105,116^ GSK2850163 is a screening-identified selective IRE1 inhibitor that bound to the kinase domain and displaced the kinase activation loop, thereby inhibiting RNase activity with an IC_50_ of 200 nM.^117^ Compound **31** was further identified as an IRE1-selective inhibitor (IC_50_ 80 nM) that stabilized the inactive kinase conformation allosterically.^118^ Unfolded protein response inhibitor UPRM8 is a covalent inhibitor targeting the IRE1 kinase domain of IRE1 by reacting to a conserved cysteine residue in the active site.^119^ In contrast to the type II kinase inhibitors, type I kinase inhibitors activated the RNase domain of IRE1. Examples of such activators with poor selectivity include the receptor tyrosine kinase inhibitor sunitinib and the promiscuous kinase inhibitor APY29.^97^ In contrast, the pyrazolopyridine compound G-1749 activated unphosphorylated IRE1 via the modulation of the activation loop with an EC_50_ below 0.1 μM and showed a favorable selectivity profile.^108^ The RNase domain of IRE1 was directly targeted by covalent modifiers of lysine 907, such as hydroxy-aryl-aldehydes represented by compounds MKC9989.^120^ The lysine residue is not present in the same position in RNase L, making such Lysine-based covalent-targeting approach selective for IRE1. Furthermore, a docking study addressing the dimer interface of the RNase domain identified neomycin (IC_50_ 0.33 μM) as an IRE1 inhibitor.^112^

## BIFUNCTIONAL MOLECULES TARGETING RNASES

### Bifunctional Dicer Inhibitors

**Figure 7:**
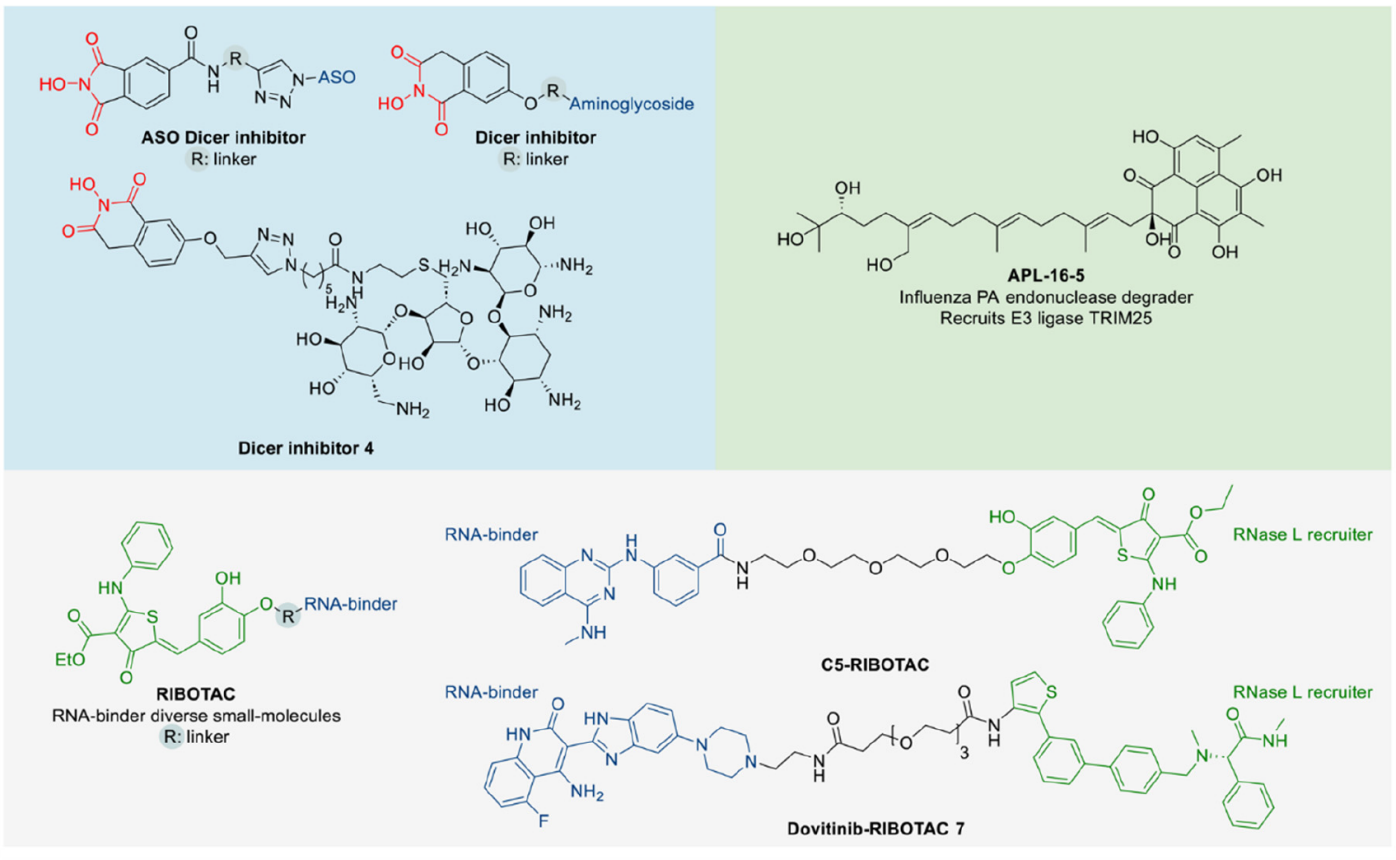
Bifunctional molecules targeting RNases. General structures and representative examples of Dicer-targeting bifunctional molecules and RNA-targeting RIBOTACs, instead of all reported examples with full structures, were shown for clarity. ASO, Antisense oligonucleotide.

An emerging approach to target ribonucleases is the design of bifunctional molecules to activate, bind, or inhibit an RNase in a specific context (Figure 7). The metal-dependent RNase Dicer is involved in the maturation of noncoding miRNAs. Bifunctional Dicer inhibitors allow specific inhibition of processing of a selected target RNA, avoiding affecting all canonically generated miRNAs by Dicer inhibition. The bifunctional inhibitors consist of an RNA-binding molecule and a weak Dicer inhibitor.^121−124^ The reported Dicer inhibitors were derived from pharmacophores of other endoribonuclease III inhibitors initially developed against influenza endonuclease and harbor the typical metal-chelating three oxygen pharmacophore.^125^ Aminoglycosides neomycin and kanamycin were employed as the RNA-binding molecules to target the oncogenic miRNA miR-21.^122,123^ The specificity of such bifunctional Dicer inhibitors was increased using antisense oligonucleotides, which alone did not inhibit the processing of pre-miR-21 by Dicer.^121^ A following study showed the possibility of incorporating a light-cleavable linker in the bifunctional molecule to deactivate the inhibitory activity by light.^124^

### PROTAC

In general, the primary type of proximity-inducing bifunctional molecules is proteolysis targeting chimeras (PROTACs) that have gained substantial attention in the past decade to achieve degradation of protein targets of interest via ubiquitination and proteasome-mediate degradation pathway.^126^ Until now, no PROTAC that degrades an RNase has been developed, while a screening hit APL-16-5 against influenza PA endonuclease was confirmed to have a PROTAC-like mechanism. APL-16-5 is a microbial metabolite of *Aspergillus sp.* CPCC 400735 with an EC_50_ of 0.28 μM in antiviral assays that was shown to prevent the lethality of influenza infections in mice. Evaluation of its mode of action revealed the gluing mechanism of binding to the PA endonuclease and the E3-ligase TRIM25 to induce ubiquitination and thus endonuclease degradation.^127^

### RIBOTACs

In comparison to the protein-degrading PROTACs, proximity-inducing bifunctional molecules have been reported by the Disney lab to induce targeted degradation of RNAs via the recruitment of RNase L, i.e., by ribonuclease targeting chimeras (RIBOTACs). So far, RNAs with different structured elements have been successfully degraded via RIBOTACs that recruit RNase L.^92,96,128−134^ The pioneering type of RIBOTACs used 2′-5′-linked oligoadenylates, the natural RNase L activator, as the RNase L recruiting component to be coupled to a dimeric *miR-96* binder. The bifunctional molecule reduced amounts of mature *miR-96* in cells.^128^ Small-molecule-based RIBOTACs using the aminothiophenone compound C1-3 as the RNase L recruiter were then reported to induce *miR-21* degradation and led to reduced metastasis in mice breast cancer models.^92^ The same RNase L recruiter was used for the design of RIBOTACs degrading oncogenic *miR-17*, *miR-18a*, and *miR-20a* cluster, degrading SARS-CoV-2 attenuator hairpin involved in frameshifting of the ribosome (C5-RIBOTAC), for isoform-specific targeting of the mRNA of quiescin sulfhydryl oxidase 1 isoform a, and degrading an expanded G4C2 RNA repeat which is associated with amyotrophic lateral sclerosis and frontotemporal dementia.^129−131,133^ Small-molecule RNA binders that do not convey biologically active interactions were conjugated to the same aminothiophenone RNase L recruiter to form RIBOTAC degraders targeting oncogenic pre-*miR-155*, *JUN* mRNA and *MYC* mRNA.^134^ Recently, a biphenyl RNase L activator identified via screening of a DNA-encoded library was used in building the dovitinib-RIBOTAC **7** that degraded *miR-21* and deactivated a *miR-21*-mediated cancer circuit in MDA-MB-231 cells.^96^ Apart from RNase L recruiters, bifunctional molecules with a chemical degrader of RNA including imidazole or a bleomycin derivative were developed.^135−137^

Both small-molecule RNase L recruiters and the Dicer inhibitor that were used as the building blocks for the bifunctional molecules are weak activators or inhibitors of their target RNases. The bifunctional molecule approach allows the local increase of RNase concentration or a local modulation of the RNase, affecting only the target RNA of interest.

## SUMMARY AND PERSPECTIVES

RNases are ubiquitous RNA-cleaving and modifying proteins that regulate RNA biology and metabolism. RNases play essential roles in various cellular functions, including immune responses, antiviral pathways, apoptosis, and inflammation reactions. Therefore, RNases have been the targets for the development of small-molecule modulators for both biological and therapeutic applications. To date, small-molecule inhibitors of bacterial, viral, and human RNases have been reported, together with limited examples of small-molecule activators targeting a few selected human RNases. Successful examples, such as the approved agent baloxavir marboxil that inhibits the influenza PA endonuclease indicate the potential to develop RNase-targeting small molecules for therapeutic purposes. Most addressed ribonucleases are metalloenzymes, and thus, the three-oxygen pharmacophore discussed in the above-mentioned metal chelating inhibitors is highly abundant. The prodrug approach of baloxavir marboxil allows for higher cellular availability and thus provides a feasible approach to yield molecules with improved effectivity. The prodrug design to shield the three-oxygen pharmacophore of many RNase-targeting small molecules can potentially improve the limited cellular activity of many reported inhibitors and will be useful for discovering further optimized molecules. It is noteworthy that many reported RNase-targeting small molecules feature structures that may interfere with assay readouts,^13^ the same applies for small-molecule inhibitors of RNA-binding proteins in general.^138^ Therefore, careful validations via orthogonal assays and evaluations on the targeting profile and mechanisms of inhibition/activation are needed to identify robust molecules to be studied as either drug candidates or useful probes. In addition to synthetic small molecules, aminoglycosides such as neomycin were repeatedly described as inhibitors against a broad range of different RNases with generally low activity and selectivity, and aminoglycosides are also known to bind to RNAs. RNase L is one of the few RNase targets for which both inhibitors and activators have been reported by targeting different structural domains. Covalent inhibitors binding to the ribonuclease or adjacent domains were reported for IRE1. Reported covalent compounds showed improved efficiency in targeting shallow binding sites and thus hold great potential for developing potent RNase-targeting small molecules.^120,139−141^ Of note, cytotoxic RNases such as Ranpirnase and Barnase have also been studied for their direct use as anticancer agents in gene therapy.^142,143^ A new perspective in the field is the emerging class of bifunctional molecules that target RNases Dicer and RNase L for specific inhibition or activation of the RNases, achieving either inhibition of RNA biogenesis or induced RNA degradation. Concerning the biological relevance of RNases and their close associations with pathological states, it is reasonable to expect that more RNases will be subject both as the protein targets of interest for bifunctional molecules and as the effector protein components to be utilized by bifunctional molecules. These RNase-targeting molecules will contribute to probe an improved understanding of RNase biology at both the transcriptional and translational levels and provide new perspectives in developing small-molecule-based therapeutics.

## Supporting Information Available

Summary of molecular weights of depicted small molecules and bifunctional molecules (PDF)

## ABBREVIATIONS

AGS: Aicardi-Goutières syndrome
BTDBA: 4-[5-(benzoylamino)thien-2-yl]-2,4-dioxobutanoic acid
CC_50_: Half maximal cytotoxic concentration
Covid: Coronavirus disease
EC_50_: Half maximal effective concentration
ENCODE: Encyclopedia of DNA Elements
FDA: United States food and drug administration
FRET: Förster resonance energy transfer
HAART: Highly active antiretroviral therapy
HBV: Hepatitis B virus
HIV: Human immunodeficiency virus
IC_50_: Half maximal inhibitory concentration
IRE1: Serine/threonine-protein kinase/endoribonuclease IRE1
PA: Polymerase acidic
PAGE: Polyacrylamide gel electrophoresis
PAINS: Pan-assay interference compounds
PARN: Poly(A)-specific ribonuclease
PCR: Polymerase chain reaction
PKR: Interferon-induced, double-stranded RNA-activated protein kinase
RIBOTAC: Ribonuclease targeting chimera
RT-PCR: Real-time polymerase chain reaction
SAMDI: self-assembled monolayers for matrix-assisted laser desorption ionization
SAR: Structure–activity relationship
SARS-CoV-2: Severe acute respiratory syndrome coronavirus 2
SPR: Surface plasmon resonance
tRNA: tRNA
XBP1: X-box binding protein-1
**KEYWORDS**
Bifunctional molecule: a molecule with two functional entities. Synthetic heterobifunctional molecules often incorporate a linker structure between the two functional entities (e.g., two small molecules) interacting with two different biomacromolecules or domains
DEDD motif: a common motif in nucleases consists of the four acidic amino acids Asp, Glu, Asp, and Asp, coordinating two divalent metal ions that coordinate the substrate phosphate in the active site of the nuclease
noncoding RNAs: RNA molecules that are not translated into proteins but usually play important regulatory roles in various biological processes and diverse cellular activities
PROTAC: proteolysis targeting chimeras are bifunctional molecules coupling a protein-binding moiety to an E3-ligase binder targeting a protein of interest for degradation
Proximity-inducing molecule: a molecule that induces post-translational modifications or temporal control of biological processes by bringing two biomarcomolecules (that would not normally interact) in close proximity
Ribonuclease: an enzyme that cleaves the phosphodiester bonds of ribonucleic acids
Ribonuclease L (RNase L): an antiviral interferon-induced ribonuclease that is activated depending on 2′-5′ oligoadenylates binding, resulting in cleavage and degradation of cellular RNAs
RIBOTAC: ribonuclease targeting chimeras are bifunctional molecules consisting of a functional unit that binds RNase L linked to an RNA-binding moiety. The RNase is recruited to the RNA of interest, inducing specific degradation of the RNA
