# Supplementary material for: Targeting Ribonucleases with Small Molecules and Bifunctional Molecules
Source: ACS Chem Biol. 2023 Jun 29;18(10):2101–13. doi: 10.1021/acschembio.3c00191 (PMC10594538; doi:10.1021/acschembio.3c00191)
Supplement: Supplementary file 1 — cb3c00191_si_001.pdf [file cb3c00191_si_001.pdf]

Supporting Information

Targeting ribonucleases with small molecules and bifunctional molecules

Lydia Borgelt and Peng Wu*

Chemical Genomics Centre, Max Planck Institute of Molecular Physiology, Otto-Hahn-Str. 11, Dortmund 44227, Germany

Department of Chemical Biology, Max Planck Institute of Molecular Physiology, Otto-Hahn-Str. 11, Dortmund 44227, Germany

*Email: peng.wu@mpi-dortmund.mpg.de

Table S1. Molecular weights of compounds depicted in Figures 2–7.

Index	Compound name	Structure	MW / g/mol
1	Neomycin B	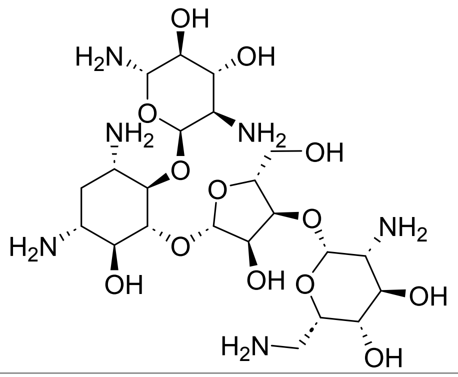	600
2	Iriginol hexaacetate	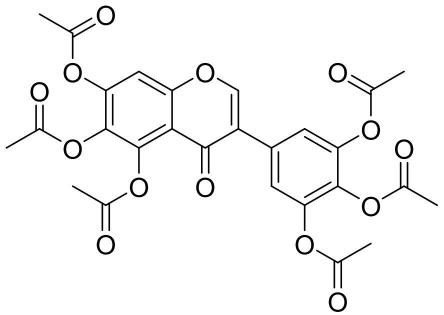	570
3	Purpurin	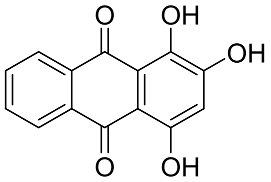	255
4	RNPA2000	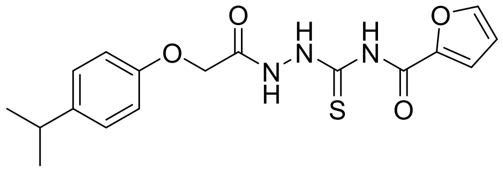	361

5	Compound **11**	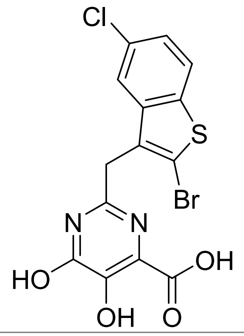	414
6	BTDBA	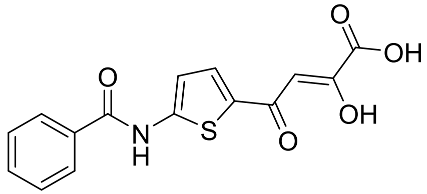	317
7	Compound **45**	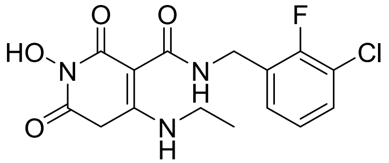	355
8	Compound **13j**	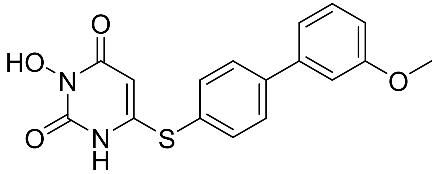	342
9	BHMP03	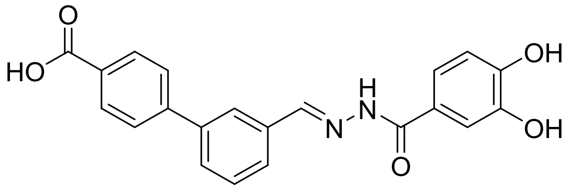	376

10	Compound **9**	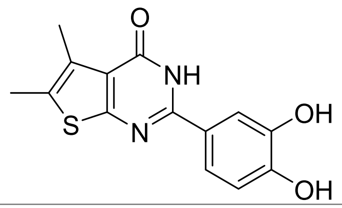	288
11	Compound **22**	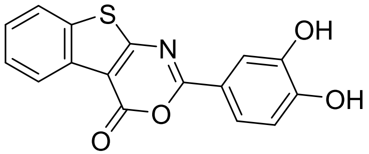	311
12	DNBNH	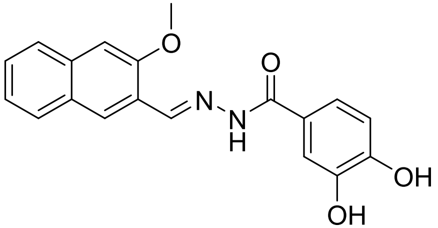	336
13	NSC95397	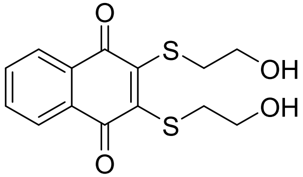	310
14	Compound #79	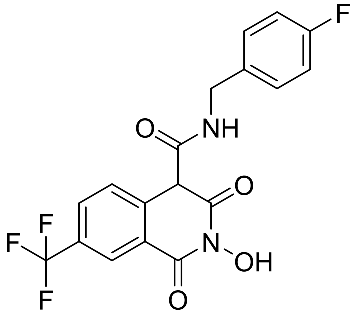	396

15	Compound 1	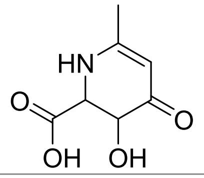	171
16	Compound 23	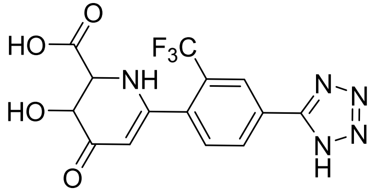	369
17	L-753,882	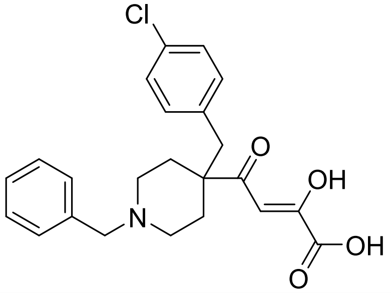	413
18	Dolutegravir	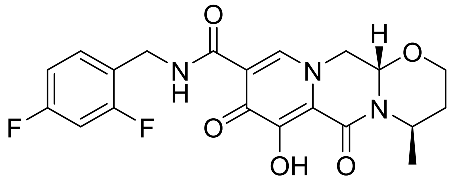	419
19	Baloxavir acid	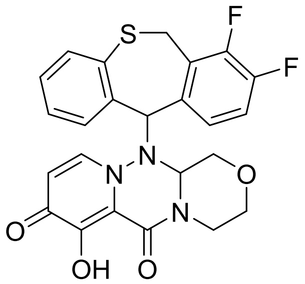	483

20	Baloxavir marboxil	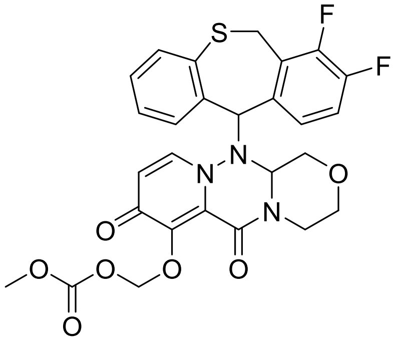	571
21	Compound **8j**	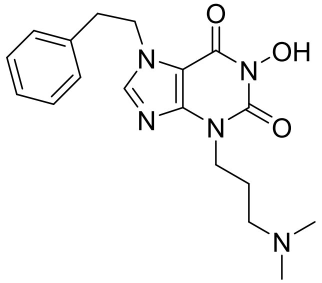	357
22	R11 / ebsulfur	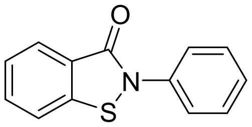	227
23	Sunitinib	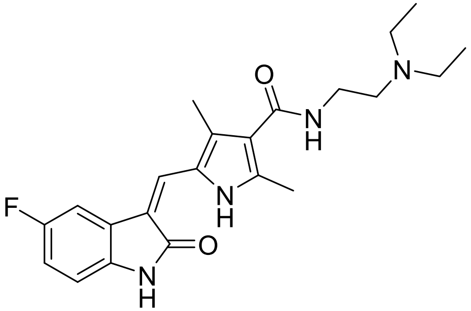	398

24	Ellagic acid	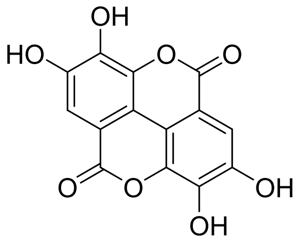	302
25	Hyperoside	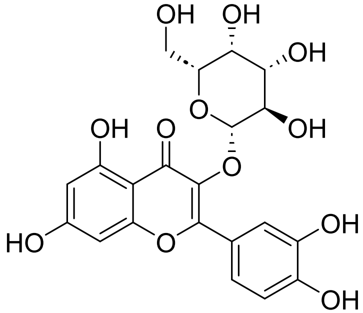	464
26	C1	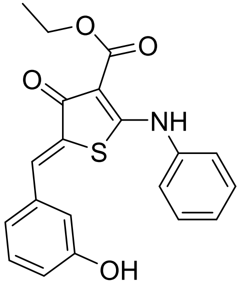	367
27	C1-3	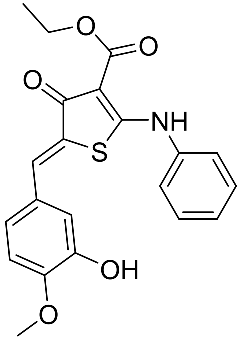	397

28	C2	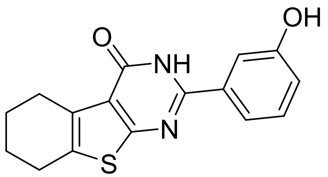	298
29	Compound **2**	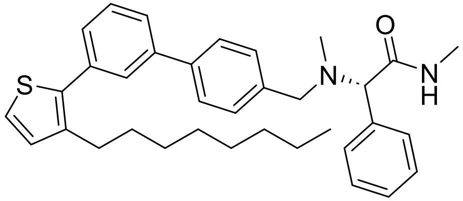	538
30	Compound **3**	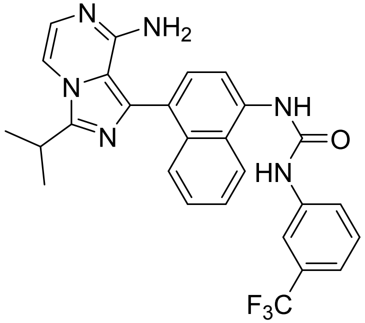	504
31	KIRA6	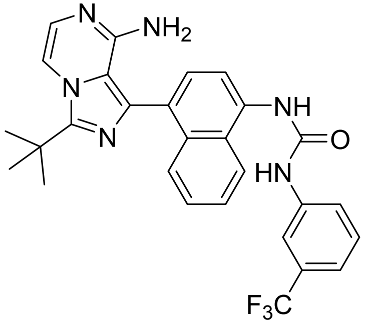	518

32	KIRA7	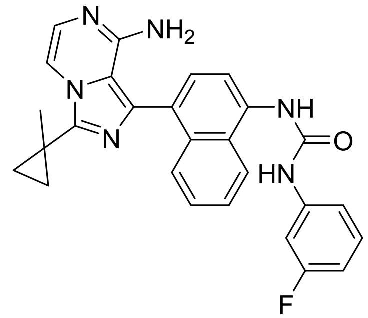	466
33	KIRA8	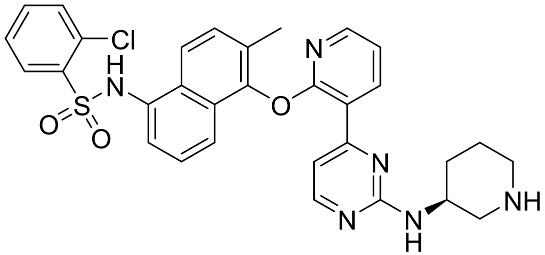	601
34	GSK2850163	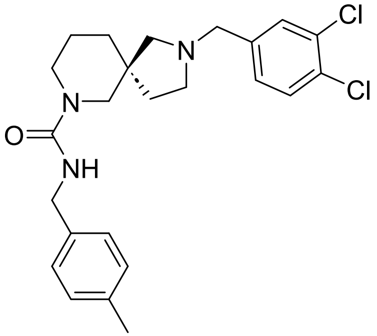	446
35	Compound **31**	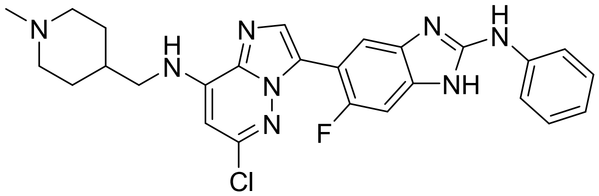	505

36	UPRM8	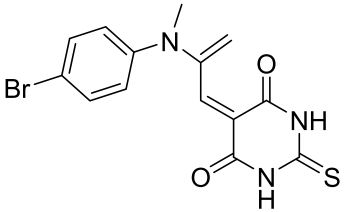	376
37	APY29	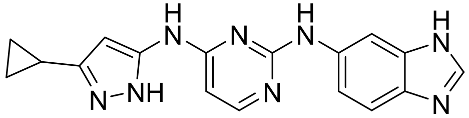	332
38	G-1749	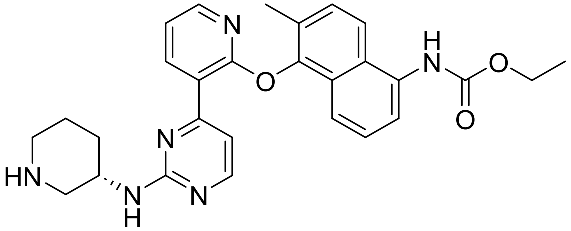	498
39	MKC9989	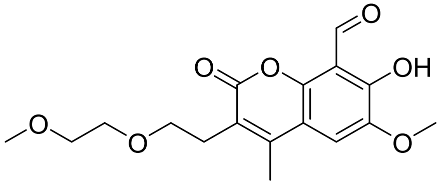	336
40	Dicer inhibitor **4**	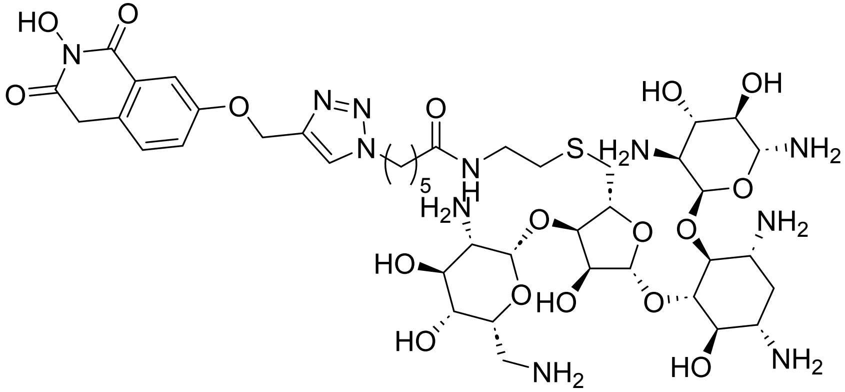	1030

41	APL-16-5	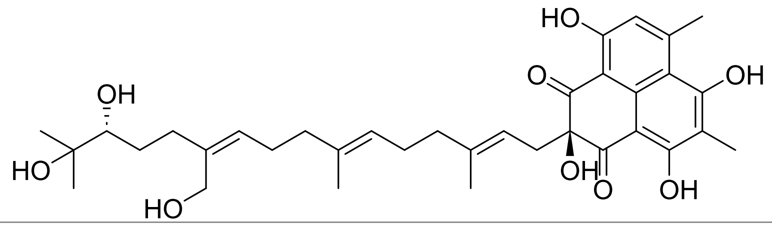	610
42	C5-RIBOTAC	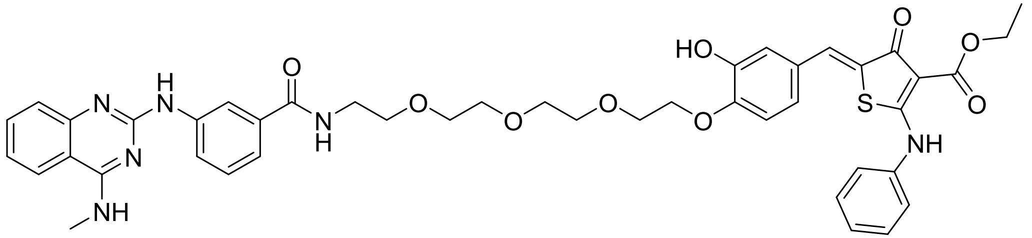	834
43	Dovitinib-RIBOTAC **7**	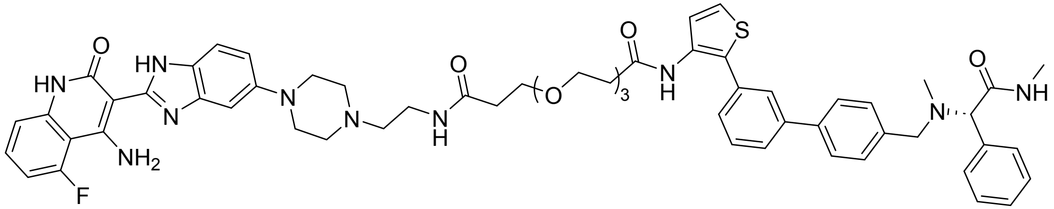	1076
